# Supplementary figures and images for: Progress towards lymphatic filariasis elimination in Ghana from 2000-2016: Analysis of microfilaria prevalence data from 430 communities
Source: PLoS Negl Trop Dis. 2019 Aug 9;13(8):e0007115. doi: 10.1371/journal.pntd.0007115 (PMC6709921; doi:10.1371/journal.pntd.0007115)

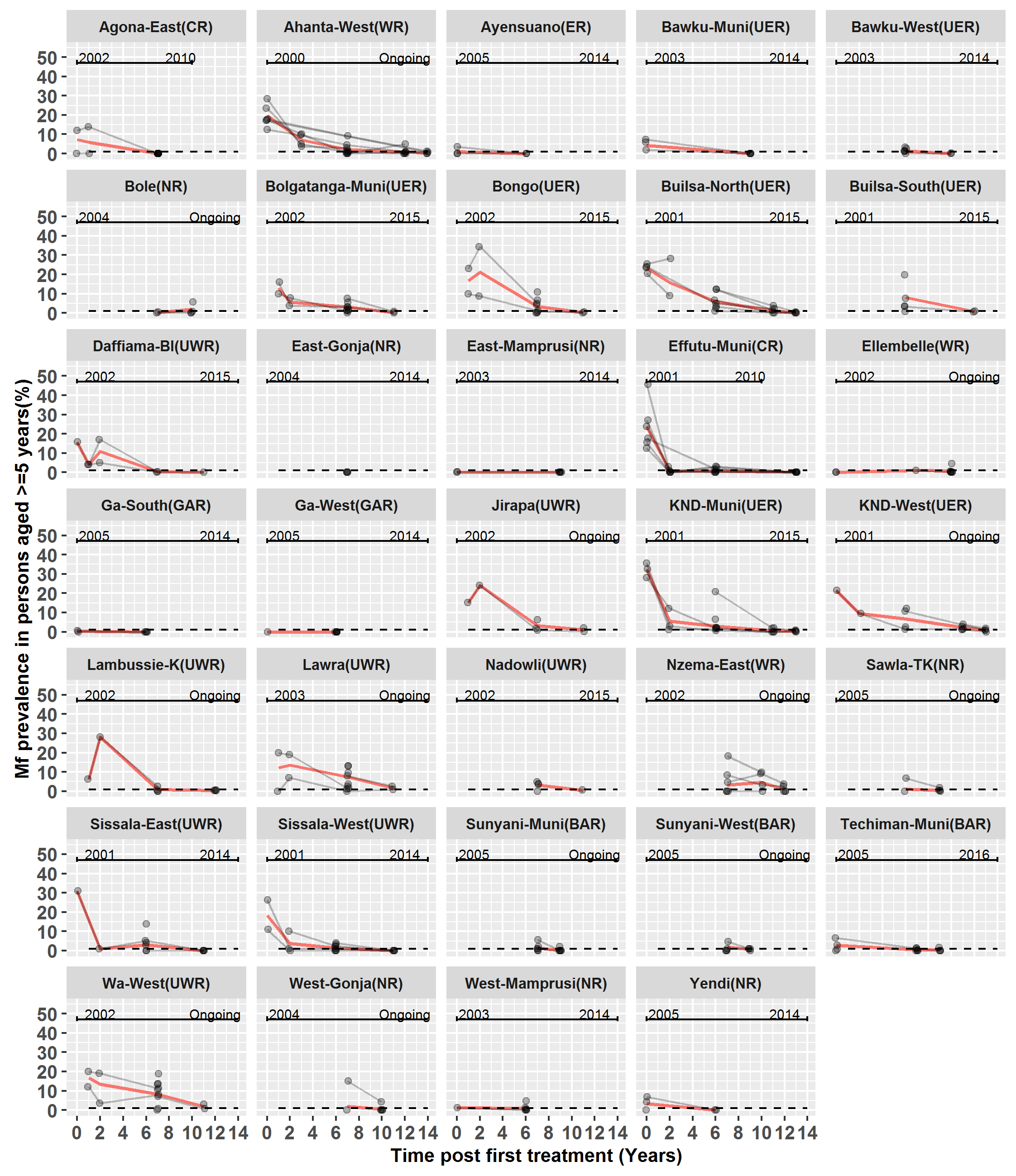

Supplement: S1 Fig — (TIF) [file pntd.0007115.s002.tif]

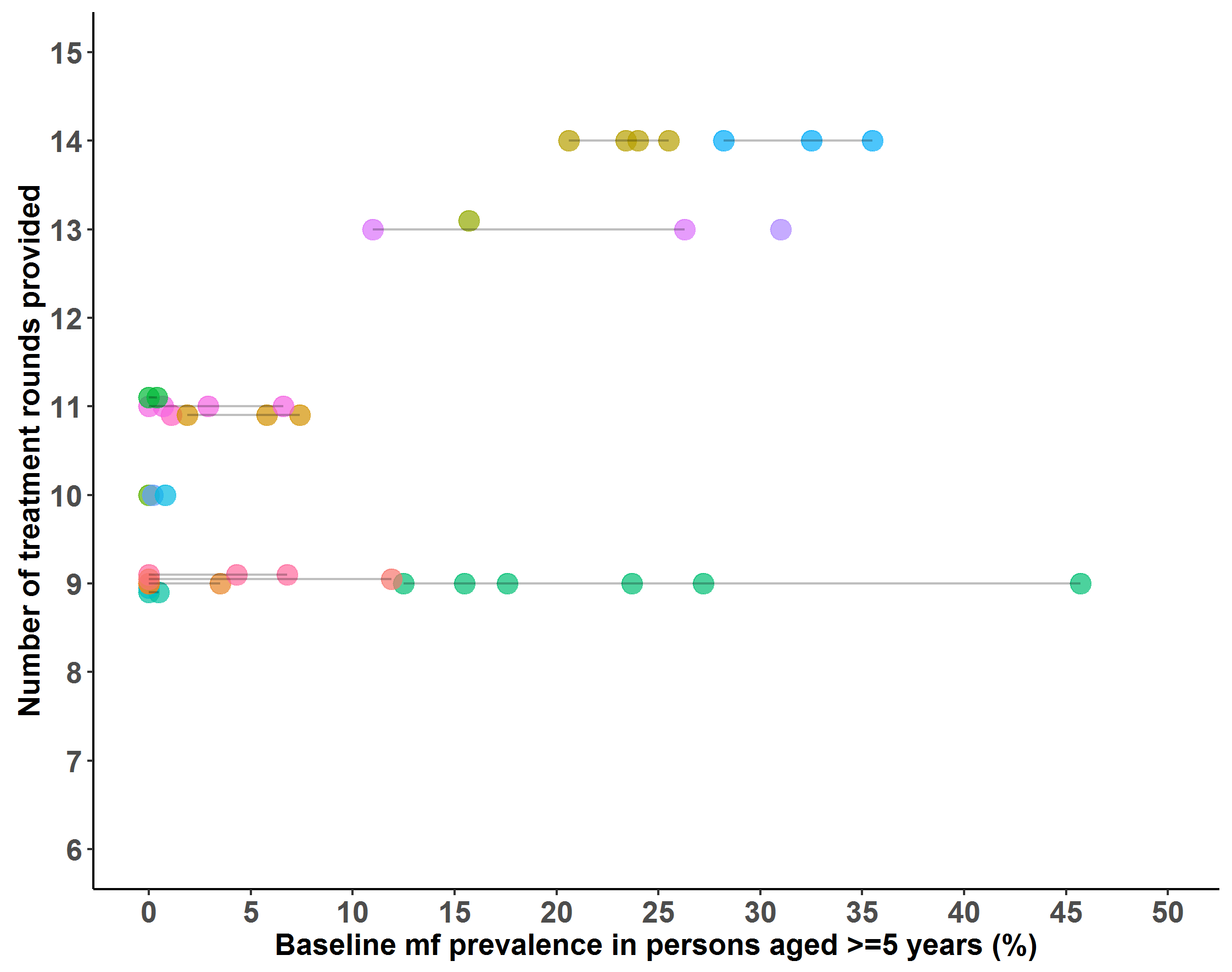

Supplement: S2 Fig — (TIF) [file pntd.0007115.s003.tif]
