# Supplementary material for: Progress towards lymphatic filariasis elimination in Ghana from 2000-2016: Analysis of microfilaria prevalence data from 430 communities
Source: PLoS Negl Trop Dis. 2019 Aug 9;13(8):e0007115. doi: 10.1371/journal.pntd.0007115 (PMC6709921; doi:10.1371/journal.pntd.0007115)
Supplement: S1 Table — (DOCX) [file pntd.0007115.s004.docx]

| **Region** | **District** | **Community** | **Year of sampling** | **No. examined** | **No. positive** | **Mf prevalence % (95%-CI)** |
| --- | --- | --- | --- | --- | --- | --- |
| Brong-Ahafo | Techiman-Municipal | Kwesi-Gyan | 2004 | 138 | 4 | 2.9 (0.1 - 5.7) |
| Brong-Ahafo | Techiman-Municipal | Nsokonee | 2004 | 151 | 10 | 6.6 (2.7 - 10.6) |
| Brong-Ahafo | Techiman-Municipal | Tandanafo-1 | 2004 | 141 | 1 | 0.7 (0 - 2.1) |
| Brong-Ahafo | Techiman-Municipal | Tandanafo-2 | 2004 | 100 | 0 | 0.0 |
| Central | Agona-East | Essusu | 2002 | 84 | 10 | 11.9 (5 - 18.8) |
| Central | Agona-East | Kwesi-Paintsil | 2002 | 52 | 0 | 0.0 |
| Central | Effutu-Municipal | Ateitu | 2000 | 103 | 16 | 15.5 (8.5 - 22.5) |
| Central | Effutu-Municipal | Atekyedo | 2000 | 72 | 9 | 12.5 (4.9 - 20.1) |
| Central | Effutu-Municipal | Gyahadze | 2000 | 92 | 42 | 45.7 (35.5 - 55.8) |
| Central | Effutu-Municipal | Gyangyanadze | 2000 | 139 | 33 | 23.7 (16.7 - 30.8) |
| Central | Effutu-Municipal | Nsuekyir | 2000 | 108 | 19 | 17.6 (10.4 - 24.8) |
| Central | Effutu-Municipal | Osubonpanyin | 2000 | 92 | 25 | 27.2 (18.1 - 36.3) |
| Central | KEEA | Ankwanda_Teterem* | 2003 | 400 | 3 | 0.8 (0 - 1.6) |
| Eastern | Ayensuano | Kofi-Pare | 2004 | 277 | 0 | 0.0 |
| Eastern | Ayensuano | Kwaboanta | 2004 | 100 | 0 | 0.0 |
| Eastern | Ayensuano | Onakwase | 2004 | 142 | 5 | 3.5 (0.5 - 6.6) |
| Greater-Accra | Ga-West | Kofi-Quaye | 2004 | 191 | 0 | 0.0 |
| Greater-Accra | Ga-South | Kudehia | 2004 | 188 | 1 | 0.5 (0 - 1.6) |
| Greater-Accra | Ga-South | Obom | 2004 | 104 | 0 | 0.0 |
| Northern | East-Gonja | Kalande_Kpembe* | 2003 | 486 | 0 | 0.0 |
| Northern | East-Mamprusi | Namaasim | 2002 | 273 | 0 | 0.0 |
| Northern | East-Mamprusi | Zaadantinga | 2002 | 230 | 1 | 0.4 (0 - 1.3) |
| Northern | West-Mamprusi | Wungu | 2002 | 441 | 5 | 1.1 (0.1 - 2.1) |
| Northern | Yendi | Adibo | 2004 | 200 | 0 | 0.0 |
| Northern | Yendi | Bumbung | 2004 | 184 | 8 | 4.3 (1.4 - 7.3) |
| Northern | Yendi | Kulkpeni | 2004 | 132 | 9 | 6.8 (2.5 - 11.1) |
| Upper-East | Bawku-Municipal | 44 | 2002 | 104 | 6 | 5.8 (1.3 - 10.3) |
| Upper-East | Bawku-Municipal | Zawsie | 2002 | 265 | 5 | 1.9 (0.2 - 3.5) |
| Upper-East | Bawku-Municipal | Ziako | 2002 | 136 | 10 | 7.4 (3.0 - 11.7) |
| Upper-East | Builsa-North | Achangyeri | 2000 | 102 | 26 | 25.5 (17.0 - 33.9) |
| Upper-East | Builsa-North | Chuchuliga-Namonsa | 2000 | 107 | 22 | 20.6 (12.9 - 28.2) |
| Upper-East | Builsa-North | Kpandema | 2000 | 121 | 29 | 24.0 (16.4 - 31.6) |
| Upper-East | Builsa-North | Pilsa | 2000 | 111 | 26 | 23.4 (15.5 - 31.3) |
| Upper-East | KND-Municipal | Biu | 2000 | 110 | 31 | 28.2 (19.8 - 36.6) |
| Upper-East | KND-Municipal | Korania | 2000 | 121 | 43 | 35.5 (27.0 - 44.1) |
| Upper-East | KND-Municipal | Namolo | 2000 | 126 | 41 | 32.5 (24.4 - 40.7) |
| Upper-East | KND-West | Baduna | 2000 | 112 | 24 | 21.4 (13.8 - 29.0) |
| Upper-West | Daffiama-BI | Touri | 2000 | 108 | 17 | 15.7 (8.9 - 22.6) |
| Upper-West | Sissala-East | Banu | 2000 | 113 | 35 | 31.0 (22.4 - 39.5) |
| Upper-West | Sissala-West | Bouti | 2000 | 109 | 12 | 11.0 (5.1 - 16.9) |
| Upper-West | Sissala-West | Sorbelle | 2000 | 99 | 26 | 26.3 (17.6 - 34.9) |
| Western | Ahanta-West | Asemasa | 2000 | 98 | 17 | 17.3 (9.9 - 24.8) |
| Western | Ahanta-West | Asemko | 2000 | 112 | 14 | 12.5 (6.4 - 18.6) |
| Western | Ahanta-West | Busua | 2000 | 124 | 29 | 23.4 (15.9 - 30.8) |
| Western | Ahanta-West | Butre | 2000 | 123 | 35 | 28.5 (20.5 - 36.4) |
| Western | Ahanta-West | Cape-3-points | 2000 | 99 | 17 | 17.2 (9.7 - 24.6) |
| Western | Ahanta-West | Mpataano | 2000 | 106 | 19 | 17.9 (10.6 - 25.2) |
| Western | Ellembelle | Anwia | 2002 | 100 | 0 | 0.0 |
| Western | Ellembelle | Bomoakpoley | 2002 | 99 | 0 | 0.0 |
| Western | Shama | Shama_Shama-Kumasi* | 2003 | 557 | 3 | 0.5 (0 - 1.1) |

*Mf prevalence data were combined from two communities in the same district. Districts names in the table represent the current districts after the re-demarcations.

Mf = microfilaria, CI = confidence interval
