# Supplementary material for: Progress towards lymphatic filariasis elimination in Ghana from 2000-2016: Analysis of microfilaria prevalence data from 430 communities
Source: PLoS Negl Trop Dis. 2019 Aug 9;13(8):e0007115. doi: 10.1371/journal.pntd.0007115 (PMC6709921; doi:10.1371/journal.pntd.0007115)
Supplement: S2 Table — (DOCX) [file pntd.0007115.s005.docx]

| **District** | **District number** | **Region** | **District population (2010)** | **Mf data available -Time post 1st treatment (year)** | **Baseline mf prevalence (range)** | **MDA start year*** | **TAS-1 (Year of last treatment)** | **Total no. of treatment rounds received by 2016** | **TAS-2 (year)** | **TAS-3 (year)** | **Hotspot (2016) MDA on-going** |
| --- | --- | --- | --- | --- | --- | --- | --- | --- | --- | --- | --- |
| Abura Asebu Kwamankese (AAK) | 28 | Central | 117,185 | 8 | - | 2003 | 2014 | 11 | 2016 | Not yet | - |
| Accra Metro | 96 | Greater Accra | 1,848,614 | 7 | - | 2006 | 2014 | 8 | 2016 | Not yet | - |
| Agona East | 11 | Central | 85,920 | 0,1 | 6 (0 - 11.9) | 2002 | 2010 | 9 | 2012 | 2015 | - |
| Agona West Municipal | 12 | Central | 115,358 | - | - | 2002 | 2010 | 9 | 2012 | 2015 | - |
| Ahanta West | 1 | Western | 106,215 | 0,3,7,12,14 | - | 2000 | Not yet | 16 | Not yet | Not yet | Yes |
| Ajumaku Enyan Essiam (AEE) | 29 | Central | 138,046 | 8 | - | 2003 | 2014 | 11 | 2016 | Not yet | - |
| Akrumfi | 30 | Central | 52,231 | - | - | 2003 | 2014 | 11 | Not yet | Not yet | - |
| Akwapim South | 71 | Eastern | 123,501 | 6 | - | 2005 | 2014 | 9 | 2016 | Not yet | - |
| Aowin | 55 | Western | 138,415 | 7 | 19.5 (12.5 - 28.5) | 2004 | 2014 | 10 | 2016 | Not yet | - |
| Asikuma Odoben Brakwa (AOB) | 56 | Central | 112,706 | 7 | - | 2004 | 2014 | 10 | 2016 | Not yet | - |
| Assin North | 57 | Central | 161,341 | - | - | 2004 | 2014 | 10 | 2016 | Not yet | - |
| Assin South | 58 | Central | 104,244 | 7 | - | 2004 | 2014 | 10 | 2016 | Not yet | - |
| Awutu Senya East Municipal | 2 | Central | 108,422 | - | - | 2001 | 2010 | 10 | 2012 | 2015 | - |
| Awutu Senya West | 3 | Central | 86,884 | 6 | - | 2001 | 2010 | 10 | 2012 | 2015 | - |
| Ayensuano | 72 | Eastern | 77,193 | 0,6 | - | 2005 | 2014 | 9 | 2016 | Not yet | - |
| Bawku Municipal | 31 | Upper East | 217,791 | 0,9 | 5 (1.9 - 5.8) | 2003 | 2014 | 11 | 2016 | Not yet | - |
| Bawku West | 32 | Upper East | 94,034 | 6,10 | - | 2003 | 2014 | 11 | Not yet | Not yet | - |
| Binduri | 33 | Upper East | 61,576 | - | - | 2003 | 2014 | 11 | 2016 | Not yet | - |
| Bole | 68 | Northern | 61,593 | 7,10 | - | 2004 | Not yet | 12 | Not yet | Not yet | Yes |
| Bolgatanga Municipal | 15 | Upper East | 131,550 | 1,2,7,11 | - | 2002 | 2015 | 13 | Not yet | Not yet | - |
| Bongo | 16 | Upper East | 84,545 | 1,2,7,11 | - | 2002 | 2015 | 13 | Not yet | Not yet | - |
| Builsa North | 7 | Upper East | 56,477 | 0,2,6,11 | 23.4 (20.6 - 25.5) | 2001 | 2015 | 14 | Not yet | Not yet | - |
| Builsa South | 8 | Upper East | 36,514 | 6,12 | - | 2001 | 2015 | 14 | Not yet | Not yet | - |
| Bunkprugu Yunyoo | 73 | Northern | 122,591 | 7 | - | 2005 | 2014 | 9 | 2016 | Not yet | - |
| Cape Coast | 59 | Central | 169,894 | 7 | - | 2004 | 2014 | 10 | 2016 | Not yet | - |
| Central Gonja | 74 | Northern | 87,877 | 6 | - | 2005 | 2014 | 9 | 2016 | Not yet | - |
| Chereponi | 34 | Northern | 53,394 | 8 | - | 2003 | 2014 | 11 | 2016 | Not yet | - |
| Daffiama Busie Issa | 17 | Upper West | 32,827 | 0,1,2,7,11 | - | 2002 | 2015 | 13 | Not yet | Not yet | - |
| East Gonja | 60 | Northern | 135,450 | 7 | 0 | 2004 | 2014 | 10 | 2016 | Not yet | - |
| East Mamprusi | 35 | Northern | 121,009 | 0,9 | 0.22 (0 - 0.4) | 2003 | 2014 | 11 | 2016 | Not yet | - |
| Effutu Municipal | 4 | Central | 68,597 | 0,2,6,13 | 23.7 (12.5-45.7) | 2001 | 2010 | 10 | 2012 | 2015 | - |
| Ellembelle | 20 | Western | 87,501 | 0,7,10 | - | 2002 | Not yet | 14 | Not yet | Not yet | Yes |
| Ga Central | 75 | Greater Accra | 117,220 | - | - | 2005 | 2014 | 9 | 2016 | Not yet | - |
| Ga East | 76 | Greater Accra | 259,668 | 6 | - | 2005 | 2014 | 9 | 2016 | Not yet | - |
| Ga South | 77 | Greater Accra | 485,643 | 0,6 | 0.27 (0 - 0.53) | 2005 | 2014 | 9 | 2016 | Not yet | - |
| Ga West | 78 | Greater Accra | 262,742 | 0,6 | 0 | 2005 | 2014 | 9 | 2016 | Not yet | - |
| Garu Tempane | 79 | Upper East | 130,003 | 7 | - | 2005 | 2014 | 9 | 2016 | Not yet | - |
| Gomoa East | 13 | Central | 207,071 | 9 | - | 2002 | 2014 | 12 | 2016 | Not yet | - |
| Gomoa West | 14 | Central | 135,189 | - | - | 2002 | 2014 | 12 | 2016 | Not yet | - |
| Gushiegu | 36 | Northern | 111,259 | - | - | 2003 | 2014 | 11 | 2016 | Not yet | - |
| Jirapa | 21 | Upper West | 88,402 | 1,2,7,11 | - | 2002 | Not yet | 14 | Not yet | Not yet | Yes |
| Jomoro | 37 | Western | 150,107 | 8 | - | 2003 | 2014 | 11 | 2016 | Not yet | - |
| Karaga | 38 | Northern | 77,706 | 8 | - | 2003 | 2014 | 11 | Not yet | Not yet | - |
| KEEA | 61 | Central | 144,705 | 7 | 0.75 | 2004 | 2014 | 10 | 2016 | Not yet | - |
| KND-Municipal | 9 | Upper East | 109,944 | 0,2,6,11,13 | 32.1 (28.1 - 35.5) | 2001 | 2015 | 14 | Not yet | Not yet | - |
| KND-West | 10 | Upper East | 70,667 | 0,2,4,6,11,13 | 21.4 | 2001 | Not yet | 15 | Not yet | Not yet | Yes |
| Kpandai | 62 | Northern | 108,816 | - | - | 2004 | 2014 | 10 | 2016 | Not yet | - |
| Kumbungu | 39 | Northern | 39,341 | 6 | - | 2003 | 2014 | 11 | 2016 | Not yet | - |
| La Dade Kotopon | 97 | Greater Accra | 183,528 | 7 | - | 2006 | 2014 | 8 | 2016 | Not yet | - |
| La Nkwantanang Madina | 80 | Greater Accra | 111,926 | - | - | 2005 | 2014 | 9 | 2016 | Not yet | - |
| Lambussie Karni | 22 | Upper West | 51,654 | 1,2,7,12 | - | 2002 | Not yet | 14 | Not yet | Not yet | Yes |
| Lawra | 23 | Upper West | 100,929 | 1,2,7,11 | - | 2002 | Not yet | 14 | Not yet | Not yet | Yes |
| Ledzokuku krowor | 98 | Greater Accra | 227,932 | 7 | - | 2006 | 2014 | 8 | 2016 | Not yet | - |
| Mamprugu Moaduri | 40 | Northern | 46,894 | 6 | - | 2003 | 2014 | 11 | 2016 | Not yet | - |
| Mfantsiman | 41 | Central | 196,563 | 8 | - | 2003 | 2014 | 11 | 2016 | Not yet | - |
| Mion | 81 | Northern | 81,812 | - | - | 2005 | 2014 | 9 | 2016 | Not yet | - |
| Mpohor | 42 | Western | 123,996 | - | - | 2003 | 2014 | 11 | 2016 | Not yet | - |
| Nabdam | 92 | Upper East | 33,826 | 8 | - | 2005 | Not yet | 11 | Not yet | Not yet | Yes |
| Nadowli | 18 | Upper West | 94,388 | 7,11 | 15.7 | 2002 | 2015 | 13 | Not yet | Not yet | - |
| Nandom | 24 | Upper West | 46,040 | - | - | 2002 | Not yet | 14 | Not yet | Not yet | Yes |
| Nanumba North | 43 | Northern | 141,584 | 6 | - | 2003 | 2014 | 11 | 2016 | Not yet | - |
| Nanumba South | 44 | Northern | 93,464 | - | - | 2003 | 2014 | 11 | 2016 | Not yet | - |
| North Gonja | 69 | Northern | 43,547 | 10 | - | 2004 | Not yet | 12 | Not yet | Not yet | Yes |
| Nsawam Adoagyiri | 82 | Eastern | 86,000 | - | - | 2005 | 2014 | 9 | 2016 | Not yet | - |
| Nzema East | 25 | Western | 60,828 | 7,10,12 | 0 | 2002 | Not yet | 14 | Not yet | Not yet | Yes |
| Prestea Huni Valley | 45 | Western | 159,304 | - | - | 2003 | 2014 | 11 | 2016 | Not yet | - |
| Pusiga | 46 | Upper East | 57,677 | - | - | 2003 | 2014 | 11 | 2016 | Not yet | - |
| Saboba | 47 | Northern | 65,706 | 8 | - | 2003 | 2014 | 11 | 2016 | Not yet | - |
| Sagnerigu | 83 | Northern | 148,099 | - | - | 2005 | 2014 | 9 | 2016 | Not yet | - |
| Savelugu Nanton | 48 | Northern | 139,283 | 6 | - | 2003 | 2014 | 11 | 2016 | Not yet | - |
| Sawla Tuna Kalba | 93 | Northern | 99,863 | 6,9 | - | 2005 | Not yet | 11 | Not yet | Not yet | Yes |
| Sekondi Takoradi metro | 63 | Western | 559,548 | 8 | - | 2004 | 2014 | 10 | 2016 | Not yet | - |
| Shama | 64 | Western | 81,966 | 7 | - | 2004 | 2014 | 10 | 2016 | Not yet | - |
| Sissala East | 5 | Upper West | 56,528 | 0,6,11 | 0.54 | 2001 | 2014 | 13 | 2016 | Not yet | - |
| Sissala West | 6 | Upper West | 49,573 | 0,2,6,11 | 31 | 2001 | 2014 | 13 | 2016 | Not yet | - |
| Suaman | 65 | Western | 20,529 | - | 18.6 (11 - 26) | 2004 | 2014 | 10 | 2016 | Not yet | - |
| Suhum | 84 | Eastern | 167,551 | 6 | - | 2005 | 2014 | 9 | 2016 | Not yet | - |
| Sunyani Municipal | 94 | Brong Ahafo | 123,224 | 7,9 | 1.2 (0 - 3.5) | 2005 | Not yet | 11 | Not yet | Not yet | Yes |
| Sunyani West | 95 | Brong Ahafo | 85,272 | 7,9 | - | 2005 | Not yet | 11 | Not yet | Not yet | Yes |
| Talensi | 89 | Upper East | 115,020 | 8 | - | 2005 | 2015 | 10 | Not yet | Not yet | - |
| Tamale Metro | 85 | Northern | 371,351 | 6 | - | 2005 | 2014 | 9 | 2016 | Not yet | - |
| Tarkwa Nsuaem | 49 | Western | 90,477 | 5 | - | 2003 | 2014 | 11 | 2016 | Not yet | - |
| Tatale Sanguli | 50 | Northern | 60,039 | 6 | - | 2003 | 2014 | 11 | 2016 | Not yet | - |
| Techiman Municipal | 90 | Brong Ahafo | 206,856 | 0,7,9 | - | 2005 | 2016 | 11 | Not yet | Not yet | - |
| Techiman North | 91 | Brong Ahafo | 59,068 | 9 | 2.6 (0 - 6.6) | 2005 | 2016 | 11 | Not yet | Not yet | - |
| Tolon | 51 | Northern | 112,331 | 6 | - | 2003 | 2014 | 11 | 2016 | Not yet | - |
| Twifo Ati Mokwa | 66 | Central | 61,743 | - | - | 2004 | 2014 | 10 | 2016 | Not yet | - |
| Twifo Heman Lower Denkyira | 67 | Central | 116,874 | 7 | - | 2004 | 2014 | 10 | 2016 | Not yet | - |
| Upper West Akim | 86 | Eastern | 87,051 | 6 | - | 2005 | 2014 | 9 | 2016 | Not yet | - |
| Wa East | 26 | Upper West | 72,074 | 11 | - | 2002 | Not yet | 14 | Not yet | Not yet | Yes |
| Wa Municipal | 19 | Upper West | 107,214 | 11 | - | 2002 | 2015 | 13 | Not yet | Not yet | - |
| Wa West | 27 | Upper West | 81,348 | 1,2,7,11 | - | 2002 | Not yet | 14 | Not yet | Not yet | Yes |
| Wassa East | 52 | Western | 81,073 | 8 | - | 2003 | 2014 | 11 | 2016 | Not yet | - |
| West Akim Municipal | 87 | Eastern | 195,349 | 6 | - | 2005 | 2014 | 9 | 2016 | Not yet | - |
| West Gonja | 70 | Northern | 84,727 | 7,10 | - | 2004 | Not yet | 12 | Not yet | Not yet | Yes |
| West Mamprusi | 53 | Northern | 168,011 | 0,6 | 1.1 | 2003 | 2014 | 11 | 2016 | Not yet | - |
| Yendi | 88 | Northern | 199,592 | 0,6 | 3.7 (0 - 6.8) | 2005 | 2014 | 9 | 2016 | Not yet | - |
| Zabzugu | 54 | Northern | 123,854 | 6 | - | 2003 | 2014 | 11 | 2016 | Not yet | - |

* All communities in each district were expected to be treated in the same year MDA started, so the geographical coverage at district level is 100% right from the start. So far, all TAS surveys were passed (no failures). The year in which TAS surveys were done, were the same year of last MDA. Districts names in the table represent the current districts after the re-demarcations. Need to explain all abbreviations here
